# The effects of anxiety on taste perception: The role of awareness

**DOI:** 10.1177/20416695231216370

**Published:** 2023-11-23

**Authors:** Naoya Zushi, Monica Perusquía-Hernández, Saho Ayabe-Kanamura

**Affiliations:** 13121 University of Tsukuba, Tsukuba, Japan; 12708 Nara Institute of Science and Technology, Ikoma, Japan; 26371 NTT Communication Science Laboratories, Atsugi, Japan; 13121 University of Tsukuba, Tsukuba, Japan

**Keywords:** taste perception, emotion, anxiety, awareness, sweetness, bitterness

## Abstract

Prior research indicate that emotional states can alter taste perception, but the underlying mechanisms remain unclear. This study explores whether taste perception changes due to the mere evocation of emotions or the cognitive awareness of emotions. The first experiment investigated how anxiety affects taste perception when individuals are aware of their anxiety. Participants watched videos inducing relaxation or anxiety, then were divided into groups focusing on their emotions and those who did not, and the taste perception was measure. The second experiment investigated the influence of awareness directed toward emotions on taste evaluation, without manipulating emotional states. This focused on cognitive processing of taste through evaluations of visual stimuli. Results showed that sweetness perception is suppressed by the evocation of anxiety, whereas bitterness perception is enhanced only by anxiety with awareness. These findings indicate that the mechanisms by which emotional states affect taste perception may differ depending on taste quality.

Taste perception can be influenced by various situational factors, even while consuming physically identical foods or drinks. An interesting illustration of such interactions in taste perceptions can be seen in cross-modal interactions, in which sensory modalities interact with and influence one another. Research has shown that visual (Moir, 1936; Piqueras-Fiszman et al., 2012; Stewart & Goss, 2013), auditory (Demattè et al., 2014; Stafford et al., 2012; Zampini & Spence, 2004), olfactory (Bonfils et al., 2005; Small & Prescott, 2005), and tactile (Biggs et al., 2016; Ichimura et al., 2023; Tu et al., 2015) stimuli can influence taste perception. Sensory information from various modalities can be integrated with gustatory information in the primary gustatory cortex after processing in several brain regions (Di Lorenzo, 2021; Liang et al., 2020; Small, 2012). This alteration in taste perception through cross-modal interaction is primarily attributed to cognitive processing; however, emotional states induced by the stimulation of other modalities may also contribute to this process (Liang et al., 2020; Spence, 2020).

Emotional states influence taste perception, and sweetness intensity is suppressed by negative emotions (Al’Absi et al., 2012; Desira et al., 2020; Dess & Edelheit, 1998; Nakagawa et al., 1996; Noel & Dando, 2015; Zushi et al., 2021). However, while one study showed that mental stress suppresses sourness intensity (Nakagawa et al., 1996), others reported that negative emotions enhance sourness intensity (Noel & Dando, 2015; Wang & Spence, 2016). Similarly, one study suggested that negative emotions (Dess & Edelheit, 1998) and music (Reinoso-Carvalho et al., 2019; Reinoso-Carvalho et al., 2020) enhance bitterness intensity, whereas another suggested that mental stress (Nakagawa et al., 1996) and intense noise (Bravo-Moncayo et al., 2020) suppress bitterness intensity. Thus, similar negative emotions have been reported to exert opposing effects on taste perception.

Our current understanding of the emotional states’ influence on taste perception is largely phenomenological; underlying mechanisms remain unclear. Two hypotheses have been proposed to explain these phenomena. First, an emotional state could potentially influence taste perception through conscious awareness; external information, such as colors or sounds, is integrated with information from taste receptors through the perception of other modalities. Similarly, our internal emotional state may also influence taste perception through awareness. A metaphorical association has been suggested between emotions and taste, with sweetness often related to positive and sourness and bitterness to negative emotions (Liang et al., 2021; Zhou & Tse, 2020). This metaphorical relationship may be reflected in the information integration during taste perception processing. If taste perception is altered by cognitive factors in such a way, this may explain the discrepancy between previous studies on sourness and bitterness. In other words, situations in which one is aware of negativity, such as watching sports (Noel & Dando, 2015), anticipation for noise as mild stressor (Dess & Edelheit, 1998), or negative music (Reinoso-Carvalho et al., 2019; Reinoso-Carvalho et al., 2020), may induce the awareness of this negativity and reflect it in the evaluation of metaphorically relevant tastes. On the other hand, a 40-min psychological task (Nakagawa et al., 1996) or intense noise (Bravo-Moncayo et al., 2020) may weaken taste by interfering with the process of the taste perception. It has also been shown that low arousal negative emotion is related to sourness and high arousal negative emotion to bitterness (Liang et al., 2021), suggesting that qualitative differences in induced emotion may account for the differences in results.

Second, emotional states can modulate taste perception at the unconscious level, irrespective of one's focus on these emotions. For example, the intake of sweet substances has been demonstrated to induce recovery from stress responses even in neonates (Smith et al., 1990), suggesting a possible innate biological significance in the relationship between sweetness and stress. Additionally, stress increases the consumption of sweet and high-fat foods (Cartwright et al., 2003; Herman & Polivy, 1975; Olive & Wardle, 1999; Oliver et al., 2000; Wardle et al., 2000). Consumption changes are complex due to the interplay of various factors (Macht, 2008), making it difficult to observe the direct influence of emotional states alone. Nevertheless, people with high stress-induced cortisol reactivity consume more sweet (Epel et al., 2001) and snack foods (Newman et al., 2007). Therefore, it is plausible that under specific circumstances, emotional states may unconsciously alter perception through physiological changes, promoting the intake of sweet substances.

In this study, two experiments were conducted to test these hypotheses. Experiment 1 examined how the influence of the emotional state on taste perception changed when focusing on one's emotional state. Specifically, after inducing emotions, we manipulated the participants’ attention toward their emotional state by having them answer either a questionnaire about their emotional state or a questionnaire related to themselves, not their emotions. After inducing emotions and manipulating the focus of awareness, beverages were used as the taste stimuli. Half of the participants evaluated the taste of unsweetened coffee, a beverage characterized by sourness and bitterness, to verify the influence of negative emotions on these two taste qualities, as previous studies reported conflicting results. The remaining half used sweetened coffee to verify the suppression of sweetness by negative emotions, as confirmed in previous studies. In Experiment 2, without emotional manipulation, we examined the influence of awareness directed toward emotions on taste evaluations. Rather than evaluating taste stimuli, we measured taste evaluation for visual stimuli. This enabled us to assess the influence of awareness directed toward emotion on taste-related cognitive processing, rather than information processing from taste receptors.

## Experiment 1

### Methods

#### Design

Experiment 1 investigated the effects of emotional state and awareness on taste perception. All participants took part in two conditions in which relaxation and anxiety were elicited by watching a 15-min video. The focus of awareness was manipulated by the type of questionnaire answered following emotion induction. Half of the participants were prompted to introspect regarding their own emotional state (emotion group: EG), and the other half were prompted to introspect about their personality traits (NEG). Thus, the influence of the induced emotions was designed as a within-subjects factor, and the focus of awareness was designed as a between-subjects factor. Additionally, half the participants evaluated the taste of an unsweetened stimulus, and the other half evaluated the taste of a sweetened stimulus (also a between-subjects factor). In summary, participants were divided into four groups based on their focus of awareness after emotional manipulation (EG/NEG) and the type of taste stimulus evaluated (unsweetened/sweetened).

#### Participants

Eighty-three Japanese participated and assigned to four groups (EG-unsweetened: *N *= 21, 11 females, *M *= 20.76 ± 1.23 years; EG-sweetened: *N *= 21, 10 females, *M *= 22.19 ± 2.54 years; NEG-unsweetened: *N *= 21, 13 females, *M *= 20.67 ± 1.39 years; NEG-sweetened: *N *= 20, 12 females, *M *= 22.05 ± 3.39 years). The sample size for each taste stimulus group was determined using G*Power 3.1 (Faul et al., 2009) in a two-way design, with the emotional condition and object of attention set as factors (*η*_p_^2^ = .17, α error = .05, 1 - β = .80). The effect size refers to the effect of anxiety on taste perception, as previously reported (Zushi et al., 2021). Because obesity has been found to be associated with changes in taste intensity (Bartoshuk et al., 2006; Hardikar et al., 2017) and sensitivity (Overberg et al., 2012; Pasquet et al., 2007; Simchen et al., 2006), only individuals with BMI less than 30 were enrolled. In addition, the participants were restricted from eating or drinking anything other than water for 2 hr prior to participation in the experiment. Participants were recruited through an Internet announcement and received a gift of instant coffee as a reward at the end of the experiment. Before and after the experiment, the participants listened to an explanation and signed a consent form.

#### Materials

Video stimuli. To induce emotional states, we used two types of videos lasting approximately 15 min each, the effects of which were confirmed in a previous study (Zushi et al., 2021). “Next Journey” (Tahara, 2010) was used for the relaxation condition, and “Dark Water” (Nakata, 2002) was used for the anxiety condition. The video in the relaxation condition showed slow sightseeing by train, and the video in the anxiety condition was an old Japanese horror film. Neither of the video contained grotesque or appetizing scenes, and each video was viewed on a personal computer (PC) screen with headphones. After watching each video, participants rated their emotional valence (0, unpleasant; 100, pleasant) and arousal level (0, calm; 100, aroused) on a visual analog scale (VAS).

Unsweetened coffee powder (Blendy®; AGC, Japan) dissolved in mineral water (SUNTORY, Japan) was used as the unsweetened stimulus. To make the sweetened stimulus, 3 g of sugar (UCC, Japan) was added. In this experiment, commercial beverages were used instead of taste solutions (e.g., citric acid and quinine solutions) to avoid influencing the evaluation of the divergence between taste stimuli and the taste experiences of everyday life. Each stimulus was aliquoted in samples of 150 ml in a clear plastic cup and maintained at 10–15°C. In this study, the same taste stimulus was evaluated twice because each participant participated in two conditions (relaxation and anxiety). For taste evaluation, the participants rated the intensity of sweetness, saltiness, sourness, bitterness, and pleasantness using a VAS ranging from 0 (very weak) to 100 (very strong).

Two questionnaires were administered to assess the participant's awareness. The EG participants responded to the STAI-JYZ (Hidano et al., 2000) questionnaire, which is the Japanese version of the State-Trait Anxiety Inventory (Spielberger et al., 1983), while NEG participants responded the new personality inventory (Yanai et al., 1987) questionnaire. The EG responded to 20 items related to state anxiety on the STAI-JYZ in the relaxation and anxiety conditions. The NEG participants responded to 20 items on each condition from the new personality inventory.

The α-amylase activity in saliva has been used as a physiological stress biomarker (Briguglio et al., 2021; Chojnowska et al., 2021; Poquérusse et al., 2018). Therefore, the saliva of each participant was measured under each condition. A salivary amylase monitor (NIPRO, Japan) was used for the measurements.

#### Procedure

**Figure 1.**
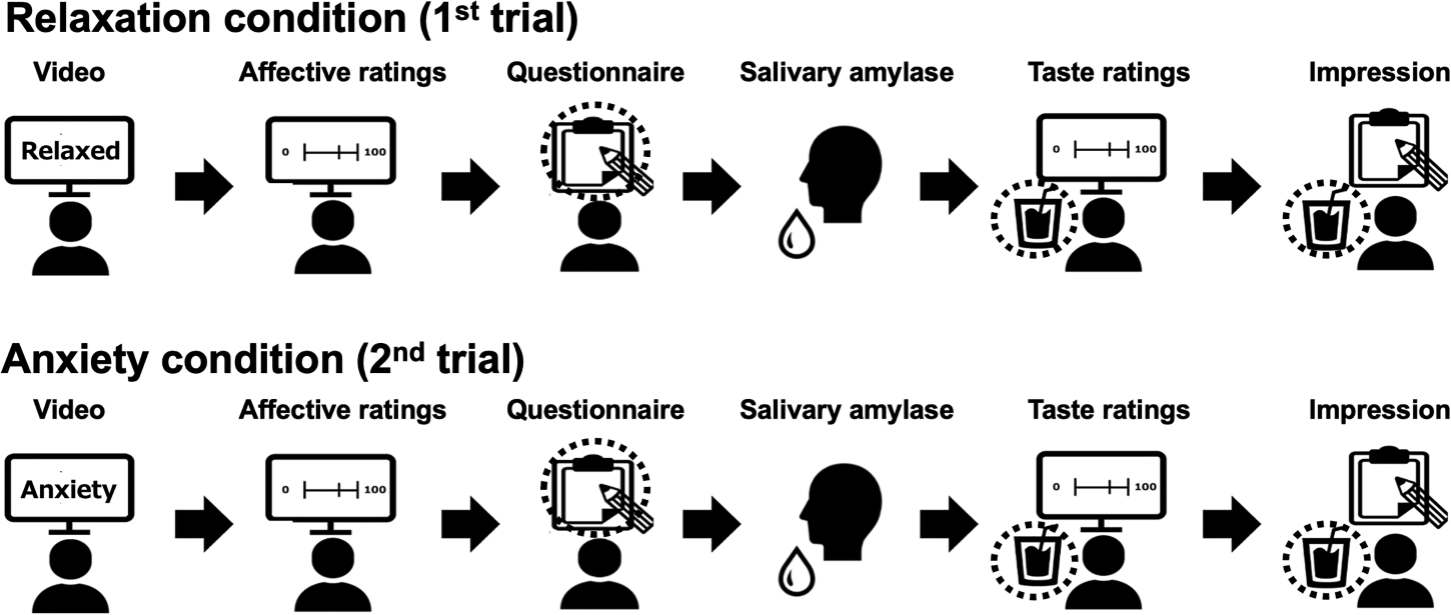
Procedure of Experiment 1. All participants performed the relaxation condition followed by the anxiety condition. The types of questionnaires and taste stimuli differed among participants.

This experiment comprised two trials (relaxation and anxiety conditions), which differed only in the video stimuli presented to the participants (Figure 1). Because it was unclear how long the impact of anxiety on taste would last, all participants first performed the relaxation condition as a control, meaning the order of the conditions was not counterbalanced. First, participants were asked to drink 100 ml of mineral water to rinse their mouth. Subsequently they wore headphones and watched the video for the relaxation conditions. Immediately after viewing the video, the participants evaluated their emotional valence and arousal levels and then answered the questionnaire assigned to their group. After answering the questionnaire, amylase activity in the saliva was measured. Next, the participants were presented with taste stimuli (unsweetened or sweetened) and asked to drink a sufficient amount to evaluate their taste, followed by evaluation of the sweetness, saltiness, sourness, bitterness, and pleasantness of the stimulus. After taste evaluation, participants were asked to report their impressions of the taste stimuli and video for 3 min. They were also informed that the consumption of the taste stimuli was voluntary. Consumption volume was then measured. After this trial, the participants were informed that a horror video would be used in the next trial and only for those who agreed to participate in the anxiety condition. The experiment was conducted in an isolated room. The experimenter remained outside the room, while the participants watched the video or answered the questionnaire. Before the experiment, the participants were informed only that they would be asked to watch the video and evaluate the taste of coffee. Therefore, the true purpose of this study was only explained to the participants after completing the experiment. This study was approved by the Human Research Ethics Committee of the University of Tsukuba (code: Tsuku 2021–50A).

#### Analysis

All analyses were performed using R version 4.0.2 (R Core Team, 2020). For valence, arousal ratings, and salivary *α*-amylase activity, we used the generalized linear mixed model (GLMM) with emotion condition (relaxation/anxiety), awareness focus (EG/NEG), and taste stimulus (unsweetened/sweetened) set as the fixed effects and participants as the random effect. For state anxiety scores in the EG only, we used a model in which awareness focus was excluded from the fixed effects. The significance of the fixed effects and interactions was calculated using a type III analysis of variance (ANOVA) with Satterthwaite's approximation. For significant effects, least squares means (LSM) were compared across groups or conditions using a *t*-test with the Satterthwaite approximation. Because the emotional evaluation and amylase measurements were performed before the intake of the taste stimulus, the effect of the taste stimulus here represents the differences in the characteristics of the participants assigned to each taste stimulus group. These analyses were performed using the “lme4” package (Bates et al., 2015) and “lmerTest” (Kuznetsova et al., 2017).

The same GLMM was used to test the fixed effects and interactions of taste evaluations (sweetness, saltiness, sourness, bitterness, and pleasantness). As a supplementary analysis, the best-fit model for each taste evaluation was determined using the “step” function of the “lme4” package. The “step” function selects and eliminates the fixed and random effects from the presented model and subsequently proposes the most appropriate model. Each *p*-value was obtained from *F*-tests calculated using Satterthwaite's approximation for fixed effects and likelihood ratio tests for random effects. In this study, five types of taste evaluations were measured. Therefore, each *p*-value was corrected by a factor of 5 using Bonferroni correction (Dunn, 1961).

Regarding consumption, the value was analyzed using the same GLMM used for taste evaluation. In addition, several studies have found that caffeine administration influences emotional state, cognitive function, and biological indicators (Klein et al., 2010; Smit & Rogers, 2000). Because the taste stimuli used in this study also contained caffeine, it is possible that caffeine intake in the first trial influenced the emotional state and taste perception in the second trial. Therefore, a Pearson's correlation analysis was conducted to examine the relationship between the consumption of taste stimuli in the first trial and the changes in emotional states and taste evaluation between the first and second trials. A *p*-value of less than .05 was considered statistically significant for all analyses.

### Results

#### Emotional Manipulation Check

An analysis was conducted using GLMM to confirm the emotional manipulation induced by the videos regardless of the awareness focus or taste stimulus. Regarding valence ratings, we found a main effect of emotion condition [*F* (1, 83) = 409.82, *p *< .0001, *η*^2^ = .99; LSM: relaxation condition = 78.9 (*SE *= 2.03), anxiety condition = 22.1 (*SE *= 2.03)]. Planned comparisons confirmed higher valence ratings in the relaxation condition than in the anxiety condition for both awareness focus and taste stimuli (all *p*-values < .0001). There was no significant effect of awareness focus, taste stimulus, or their interaction on valence ratings.

The main effect of emotion condition was also confirmed for arousal ratings [*F* (1, 83) = 273.38, *p*-values < .0001, *η*^2^ = .95; LSM: relaxation condition = 28.3 (*SE *= 2.24), anxiety condition = 78.4 (*SE *= 2.24)]. Planned comparisons revealed that higher arousal was evoked by the video in the anxiety condition than in the relaxation condition for both awareness focus and taste stimuli (all *p*-values < .0001). Moreover, we identified a significant effect of taste stimulus on arousal ratings [*F* (1, 83) = 9.61, *p *= .0026, *η*^2^ = .03]. In the relaxation condition, the EG-sweetened stimulus induced higher arousal than the EG-unsweetened stimulus [*b *= 14.50, *SE *= 6.30, *t* (174) = 2.30, *p *= .0225]. However, no significant differences were observed among the other pairs. There was no significant effect of the awareness focus or interaction.

Amylase activity was analyzed, resulting in the exclusion of seven participants from the unsweetened group due to technical monitoring errors. The results showed a significant effect of the emotion condition on amylase activity [*F* (1, 76.62) = 27.55, *p *< .0001, *η*^2^ = .73]. Compared to the relaxation condition, the LSM of amylase activity was significantly higher after the anxiety condition for both awareness focus and taste stimuli [LSM: relaxation condition = 31.1 (*SE *= 2.50), anxiety condition = 41.5 (*SE *= 2.49); all *p*-values < .0001]. We also identified a significant effect of the taste stimulus on amylase activity [*F* (1, 77.30) = 9.07, *p *= .0035, *η*^2^ = .24]. In each emotional condition, NEG-unsweetened participants showed higher amylase activity than that of NEG-sweetened participants [relaxation condition: *b *= 16.67, *SE *= 7.09, *t* (111) = 2.35, *p *= .0204; anxiety condition: *b *= 16.22, *SE *= 7.09, *t* (111) = 2.30, *p *= .0240]. No significant differences were observed among the other pairs. In addition, there was no significant effect of awareness focus or interaction on amylase activity.

**Figure 2.**
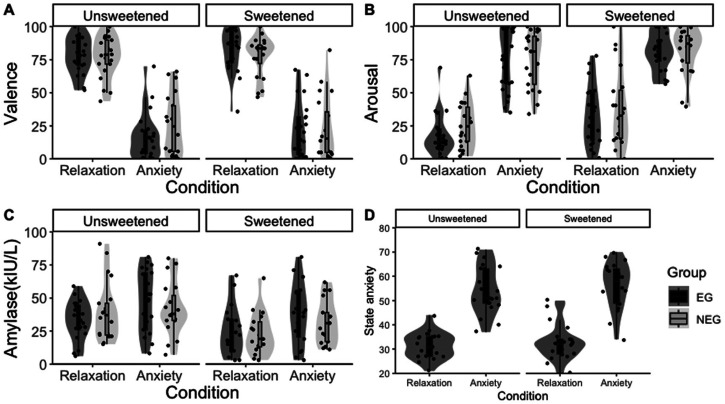
Violin and box-and-whisker plots for indicators of emotional states in Experiment 1. (A) Subjective valence ratings by emotion condition for each awareness focus group and for each taste stimulus group. (B) Subjective arousal ratings. (C) Activities of saliva *α*-amylase. (D) State anxiety scores of STAI regarding the EG participants. Comparison of LSMs confirmed significant differences by emotion condition for all indicators.

The participants’ state anxiety scores were subsequently analyzed, which revealed a significant effect of the emotion condition [*F* (1, 42) = 181.38, *p *< .0001, *η*^2^ = .99], with significantly higher state anxiety scores in the anxiety condition for both taste stimulus groups [LSM: relaxation condition = 30.9 (*SE *= 3.51), anxiety condition = 42.7 (*SE *= 3.47); all *p*-values < .0001]. No significant effect of taste stimulus or interaction between emotional condition and taste stimulus was observed on state anxiety scores. The data distribution of the emotional states is shown in Figure 2.

#### Taste Evaluations

**Figure 3.**
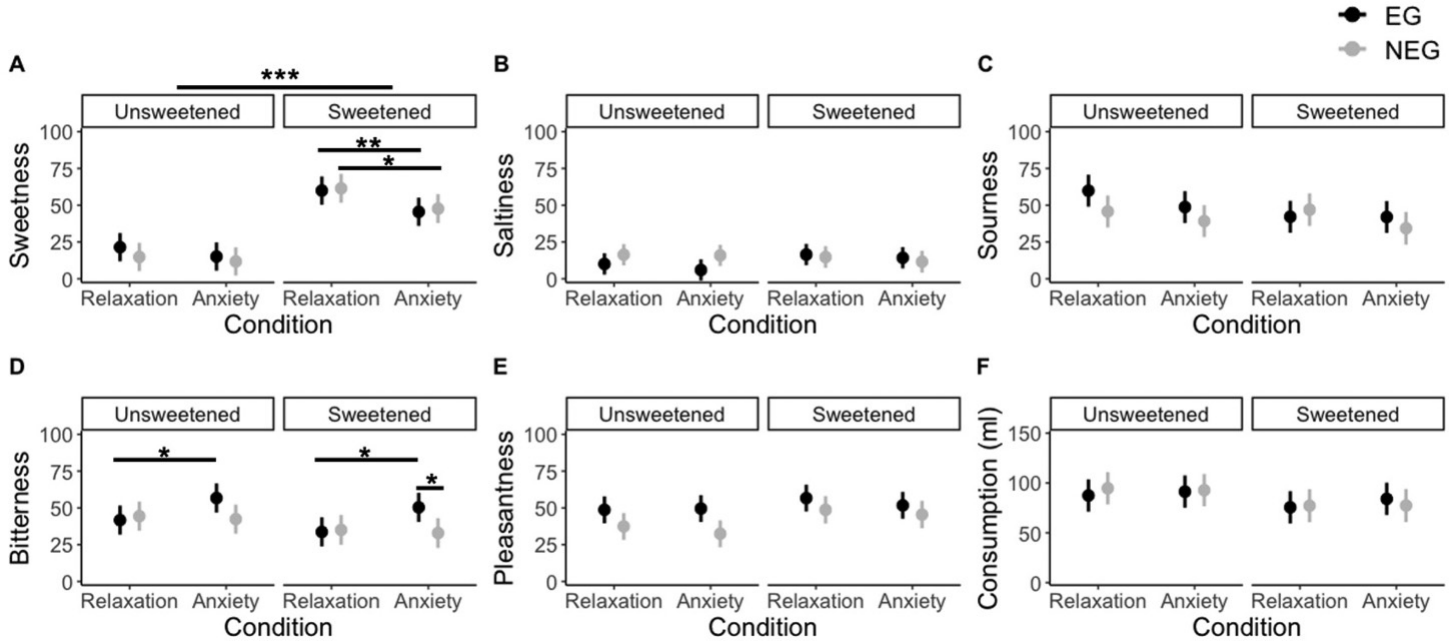
Intensity and 95% confidence interval predicted by the GLMM with emotion condition (relaxation/anxiety), awareness focus (e.g., NEG), taste stimulus (unsweetened/sweetened) as fixed effects, and participant as a random effect in Experiment 1. (A) Sweetness intensity: significant differences were found between the relaxation condition and the anxiety condition in both sweetened stimulus groups. (B) Saltiness intensity: no significant effects on saltiness were found. (C) Sourness intensity: significant differences were found between relaxation condition and anxiety condition only in the EG-unsweetened group and NEG-sweetened group. (D) Bitterness intensity: significant differences were found between the relaxation and anxiety conditions in both EGs. (E) Pleasantness was significantly influenced by taste stimulus and awareness focus. (F) Consumption: There were no significant effects on consumption. *** *p* < .001. ** *p* < .01. * *p* < .05.

The intensity ratings for each taste were analyzed using a GLMM with emotion, awareness, and taste stimuli used as the fixed effects and participants as the random effect (Figure 3). Regarding sweetness intensity, we found a significant effect of emotional condition [*F* (1, 83) = 18.07, *p *= .0003, *η*^2^ = .18]. Planned comparisons revealed a significant suppression of perceived sweetness intensity in the anxiety condition compared to the relaxation condition, regardless of awareness focus, among participants who rated the sweetened stimulus (Table 1). However, this significant difference was not observed in the group that evaluated the unsweetened stimulus. As expected, we identified a significant effect of the taste stimulus on sweetness intensity [*F* (1, 83) = 75.62, *p *< .0001, *η*^2^ = .76]. The sweetened stimulus was rated significantly sweeter than the unsweetened stimulus in both awareness focus groups under all conditions. The other fixed effects and interactions were not significant.

**Table 1.** Statistical values of comparisons between anxiety and relaxation conditions for each variable.

Variable	Stimulus	Awareness	Anxiety—relaxation			
			*b*	*SE*	*t*	*p*
Sweetness	Unsweetened	EG	−6.41	4.52	−1.42	.8020
		NEG	−3.07	4.52	−0.68	1.000
	Sweetened	EG	−14.46	4.52	−3.20	.0095
		NEG	−13.82	4.64	−3.00	.0185
Saltiness	Unsweetened	EG	−4.11	2.72	−1.51	.6720
		NEG	−0.47	2.72	−0.17	1.000
	Sweetened	EG	−2.21	2.72	−0.81	1.000
		NEG	−0.47	2.78	−1.15	1.000
Sourness	Unsweetened	EG	−11.11	4.90	−2.72	.1285
		NEG	6.57	4.90	1.34	.9175
	Sweetened	EG	−0.18	4.90	0.04	1.000
		NEG	−12.65	5.02	−2.52	.6750
Bitterness	Unsweetened	EG	15.00	5.31	2.82	.6750
		NEG	−2.01	5.31	−0.38	1.000
	Sweetened	EG	16.70	5.31	3.15	.0115
		NEG	−2.15	5.44	−0.39	1.000
Pleasantness	Unsweetened	EG	0.85	4.21	0.20	1.000
		NEG	−4.98	4.21	−1.17	1.000
	Sweetened	EG	−4.93	4.21	−1.18	1.000
		NEG	−3.22	4.31	−0.75	1.000
Consumption	Unsweetened	EG	3.91	5.40	0.72	.4712
		NEG	−1.92	5.40	−0.36	.7230
	Sweetened	EG	8.48	5.40	1.57	.1197
		NEG	0.18	5.53	0.03	.9746

In addition, we found a significant effect of emotion condition on sourness intensity [*F* (1, 83) = 18.07, *p *= .0105, *η*^2^ = .53]. However, the results of the LSM comparison showed that none of the corrected *p*-values indicated significant differences. There was no significant effect of awareness focus or taste stimulus and no interaction with sourness intensity.

Furthermore, we identified a significant main effect of emotional condition [*F* (1, 83) = 6.98, *p *= .0491, *η*^2^ = .28] and a significant interaction between emotional condition and awareness focus [*F* (1, 83) = 11.83, *p *= .0046, *η*^2^ = .47] on bitterness intensity. A comparison of LSM revealed that only EG perceived significantly stronger bitterness in the anxiety condition than in the relaxation condition. In addition, the bitterness intensity for each emotional condition was compared between the awareness focus groups. In the relaxation condition, there were no significant differences between the EG and NEG for either the unsweetened or sweetened stimuli, whereas in the anxiety condition, the EG group rated the sweetened stimulus as significantly more bitter than did the NEG group [*b *= 17.47, *SE *= 7.36, *t* (143) = -2.38, *p *= .0188]. The differences between groups for unsweetened stimuli were not significant at the 5% level [*b *= 14.35, *SE *= 7.26, *t* (143) = 1.98, *p *= .0502]. Regarding the saltiness intensity and pleasantness, none of the fixed effects or interactions showed a significant effect.

#### Consumption

Consumption was also analyzed using a GLMM with emotion conditions, awareness focus, and taste stimulus as fixed effects and participants as random effects (Figure 3). The results showed that none of the fixed effects were statistically significant. Pearson's correlation analysis was conducted to examine the influence of caffeine intake in the first trial on the emotional state in the second trial. The results revealed no significant correlation between consumption in the first trial and changes in valence (*r *= .06, *p *= .6089), arousal (*r *= -.10, *p *= 3920), amylase (*r *= -.04, *p *= .7498), or state anxiety score (*r *= -.14, *p *= .3847) in the second trial. In addition, no significant correlations with consumption in the first trial were identified for any taste evaluation changes (sweetness: *r *= .18, *p *= .1100; saltiness: *r *= -.13, *p *= .2336; sourness: *r *= -.03, *p *= .8042; bitterness: *r *= -.03, *p *= .8137; pleasantness: *r *= .03, *p *= .7845).

#### Best-Fitting Models

The best-fitting model was determined to predict each taste evaluation and consumption using the “step” function, with emotion condition (relaxation/anxiety), awareness focus (EG/NEG), and taste stimulus (unsweetened/sweetened) set as the fixed effects and participants set as a random effect. The determined models and the significance of the influence of the fixed effects, interactions, and random effects are presented in Table 2.

**Table 2.** Fixed and random effects of the best-fitting GLMM to predict each variable.

Variable	Fixed effect	Random effect
Sweetness	Emotion*** + stimulus*** + emotion × stimulus	Participant***
Saltiness		Participant***
Sourness	Emotion	Participant***
Bitterness	Emotion* + awareness + emotion × awareness**	Participant***
Pleasantness		Participant***
Consumption		Participant***

*Note*. *p < .05. **p < .01. ***p < .001.

### Discussion

In this study, the results of the emotional manipulation check confirmed that anxiety caused significantly lower valence and higher arousal and salivary amylase activity than relaxation, regardless of the awareness focus. Additionally, we found differences in arousal levels and salivary amylase activity based on the taste stimulus group. However, the interaction between the taste stimulus and emotional condition was not significantly affected. Furthermore, caffeine consumption in response to taste stimuli had no significant effect on emotional manipulation. In summary, we found that participants were significantly more anxious in the anxiety condition than in the relaxation condition, regardless of the introspection target and taste stimuli.

Emotional condition, taste stimulus, and their interaction exerted significant effects on sweetness intensity. Because the taste stimuli used in this study were unsweetened and sweetened coffee, unsurprisingly, the type of taste stimulus reflected was sweetness intensity. Meanwhile, in the sweetened stimuli evaluation groups, anxiety suppressed sweetness intensity regardless of the awareness focus. Because this effect was not observed in other taste evaluations, it can be assumed that the decrease in sweetness intensity in the anxiety condition was not due to habituation. In agreement with our findings, numerous prior studies have shown that anxiety and negative emotions suppress sweetness (Al’Absi et al., 2012; Desira et al., 2020; Dess and Edelheit, 1998; Nakagawa et al., 1996; Noel & Dando, 2015; Zushi et al., 2021). The present study revealed that the same sweetness suppression by anxiety occurs regardless of whether the participants introspect on their emotional states. Sweetness perception helps in the detection of energy sources and is so important that it is acquired innately (Mennella & Beauchamp, 1998). Indeed, significant energy is required to overcome negative situations. Therefore, the perception of sweetness may be suppressed to increase energy intake. Indeed, research has shown that the consumption of sweet and high-fat foods increases under stress (Cartwright et al., 2003; Herman & Polivy, 1975; Olive & Wardle, 1999; Oliver et al., 2000; Wardle et al., 2000). Furthermore, a high cortisol response due to stress has been shown to increase intake of sweet foods (Epel et al., 2001) and snacks (Newman et al., 2007). A study in mice also showed that glucocorticoid receptors exist in taste buds, specifically localized to Tas1r3, the taste receptor subunit for sweetness and umami, and that restraint stress induces their localization to the nucleus (Parker et al., 2014). Therefore, it can be assumed that physiological changes, such as cortisol levels, due to the negative state suppress the perceived sweetness intensity, thereby promoting energy intake.

In the present study, anxiety enhanced bitterness intensity only in the group that focused on the emotional state. Three studies have previously reported that negative stimuli can enhance bitterness intensity (Dess and Edelheit, 1998; Reinoso-Carvalho et al., 2019; Reinoso-Carvalho et al., 2020). These studies induced negative emotions using horn sounds, and music was rated as negative. However, the changes in the emotional state of the participants induced by these stimuli were not measured. In particular, in the study by Reinoso-Carvalho et al. (2019), it is unclear whether sufficient emotional arousal was achieved, as they used only a minute of music as a stimulus. However, it can be inferred that at the very least, participants’ awareness was directed toward negative stimuli during the evaluation of taste stimuli. Other studies have reported a metaphorical association between negative emotions and bitterness (Liang et al. 2021; Zhou and Tse, 2020). Integrating these findings, the enhancement of bitterness by negative emotions may result from cognitive processing effects similar to those of cross-modal influences. In other words, rather than a change in sensitivity mediated by physiological alterations, the evaluation of bitterness, which is metaphorically associated with negative emotions, is reflected by a focus on negative information in the cognitive processing of taste. Given that bitterness is crucial for detecting toxic substances (Drewnowski & Gomez-Carneros, 2000; Nissim et al., 2017), it can be argued that it is safer for changes in perceptual intensity caused by emotional states to occur solely through cognitive processes, rather than through changes in sensitivity at the peripheral level. Conversely, it has also been reported that bitterness intensity is suppressed following psychological stress (Nakagawa et al., 1996) and loud noises (Bravo-Moncayo et al., 2020). In these studies, participants were likely to be in a negative emotional state, but the observed effects on bitter perception were contrary to the results of this and other previous studies. This can be interpreted as participants’ attention to taste evaluation itself being diminished in these prior studies. Other research has shown that loud noise suppresses the intensity of taste perception (Rahne et al., 2018; Woods et al., 2011). In fact, both bitterness and the intensity of sweetness and sourness were suppressed in a stress task (Nakagawa et al., 1996), while sweetness, sourness, flavor intensity, and flavor-liking were all suppressed under loud noise (Bravo-Moncayo et al., 2020). Therefore, the contrasting changes in bitterness intensity can be interpreted as a result of attenuation of attention allocation to taste evaluation due to fatigue from a 40-min psychological task (Nakagawa et al., 1996) or loud noise that excessively draws attention (Bravo-Moncayo et al., 2020).

Furthermore, emotional condition had a significant influence on sourness intensity. The fact that a negative emotional state suppresses sourness perception is consistent with previous study results showing that mental stress suppresses sourness perception (Nakagawa et al., 1996). However, the corrected *p*-values did not differ significantly between the groups, indicating that the effect may not be robust. Other studies have reported that negative emotions enhance sourness perception (Noel & Dando, 2015; Platte et al., 2013). A possible reason for the discrepancy between studies on the influence of negative emotional states on sourness perception could be the differences in taste stimuli. Nakagawa et al. (1996) and Platte et al. (2013) used citric acid as a sourcing stimulus, but the changes in sourness perception were different. Sourness is a taste quality that is attractive at low concentrations but unpleasant at higher concentrations (Ganchrow et al., 1983; Lindemann, 2001). In addition, some individuals are receptive to high concentrations of sourness, even as infants (Blossfeld et al., 2007) and children (Liem & Mennella, 2003). The main role of sourness is the detection of acids in spoiled food, and it is therefore considered a negative taste quality; however, it also has the aspect of a positive taste quality that promotes the intake of acid to balance the concentration of acids and bases in the body (Lindemann, 2001). Thus, sourness is a more biologically complex taste quality than the others; therefore, the influence of emotional states on perception may also be complex.

In the present study, the EG showed higher pleasantness than the NEG for both emotional conditions and taste stimuli, although the corrected *p*-values did not show significance. This may be because coffee, especially unsweetened coffee, is not necessarily consumed for physically rational reasons such as energy or nutrition but is preferred to satisfy the mind. Therefore, it is possible that the group conscious of their emotional states preferred them more. However, as previously noted, this result was not sufficiently robust to be significant.

Finally, all the optimal GLMM that predicted each taste evaluation proposed in the supplementary analyses supported the above considerations.

## Experiment 2

### Methods

#### Design

In Experiment 1, the enhancement of perceived bitterness due to anxiety was suggested to reflect the metaphorical association between negative emotions and bitterness in taste evaluation. Therefore, in Experiment 2, we examined whether manipulating the emotion to which awareness was directed would influence taste evaluation without manipulating the emotional state. Here, by measuring taste evaluations in response to visual stimuli, we aimed to investigate the influence of emotional awareness on cognitive processing related to taste, but not the influence on the functioning of taste perception in the peripheral level. In particular, taste evaluations associated with the visual stimuli were compared among the three groups, in which the focus of awareness was positive emotion, negative emotion, or no emotion.

#### Participants

A total of 106 Japanese, different from those who participated in Experiment 1, took part in this experiment. Participants were assigned to the positive before evaluation group (PBE: *N* = 35, 27 females, *M* = 20.66 ± 0.95 years), the negative before evaluation group (NBE: *N* = 36, 28 females, *M* = 20.76 ± 0.85 years), and the control group (*N* = 35, 27 females, *M* = 20.17 ± 0.90 years). This sample size of each group met the required sample size calculated by G*Power 3.1 (Faul et al., 2009) with an *α* error of .05, 1 - *β* of .80, and effect size *f* of .40.

#### Materials

**Figure 4.**
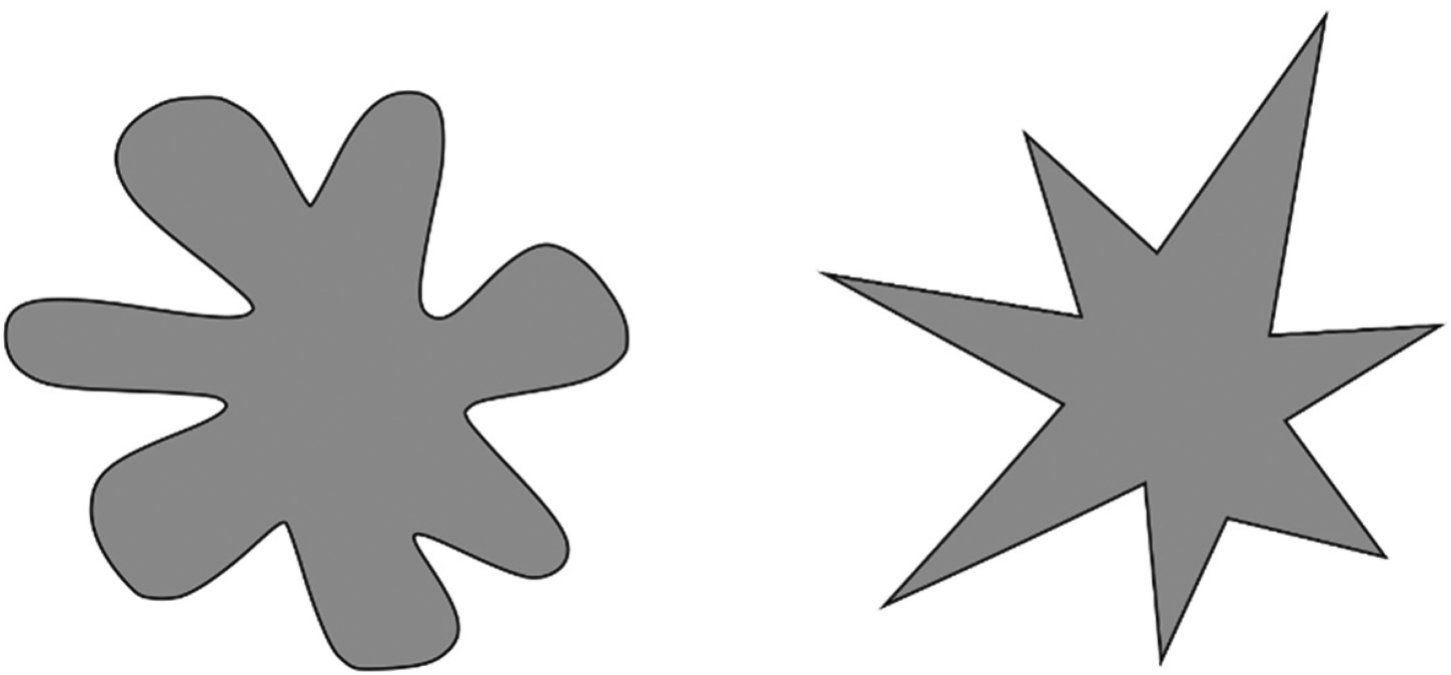
Two types of shapes subjected to taste evaluation. The one on the left is known as Bouba and the one on the right as Kiki (Ramachandran & Hubbard, 2001).

In Experiment 1, anxiety arousal influenced the ratings of perceived sweetness, and anxiety introspection influenced the ratings of perceived bitterness intensity. Various studies have shown that figure roundness is associated with sweetness, while angularity is associated with bitterness (Deroy & Valentin, 2011; Salgado-Montejo et al., 2015, Velasco et al., 2016; Velasco et al., 2015; Velasco et al., 2016). Therefore, to properly evaluate these two taste qualities, we used two types of abstract shapes as visual stimuli: Bouba, characterized by roundness, and Kiki, characterized by angularity (Figure 4: Ramachandran & Hubbard, 2001). Regarding the taste evaluation for these shapes, the participants rated the intensity of sweetness, saltiness, sourness, bitterness, and pleasantness using a VAS ranging from 0 (very weak) to 100 (very strong).

A scale of life events in the interpersonal and achievement domains for undergraduate students (Takahira, 1998) was used to induce awareness of positive or negative emotions immediately before taste evaluation. In this questionnaire, participants responded to whether they experienced the presented life event within the last 3 months, with 30 positive and 30 negative event items. Participants in the PBE who directed their attention to positive emotions immediately prior to taste evaluation responded to the positive event items following the negative event items, and participants in the NBE responded in the reverse order. The control group responded to 60 items from the same personality inventory as in Experiment 1 (Yanai et al., 1987), which was equal to the life event scale. After answering this scale, the participants rated their emotional valence (0 = unpleasant to 100 = pleasant) and arousal level (0 = calm to 100 = aroused).

#### Procedure

Participants first completed each questionnaire. Subsequently, the participants evaluated their valence and arousal using VASs. Finally, they evaluated the intensities of sweetness, saltiness, sourness, bitterness, and pleasantness associated with the two shapes (Bouba and Kiki) using a VAS. The order of shape evaluation was counterbalanced among the participants. Prior to the experiment, the participants were informed only about the completion of the questionnaire and shape evaluations. Therefore, the true purpose of the study was disclosed to the participants only following the conclusion of the experiment.

#### Analysis

All analyses were performed using R version 4.0.2 (R Core Team, 2020). To investigate the influence of the type of emotion to which awareness was directed on emotional states, we compared valence and arousal scores between the groups using a one-way ANOVA. The purpose of this experiment was to manipulate awareness of emotions by questionnaire type without manipulating emotional states. Therefore, Bayes’ coefficient (BF_10_) was calculated to reflect the likelihood ratio of the alternative hypothesis H1 (there is a difference in emotion ratings between groups) and the null hypothesis H0 (there is no difference in emotion ratings between groups). Therefore, the effect of questionnaire type on valence and arousal ratings was examined. This analysis was performed using the Bayes factor package (Bates et al. 2015). The intensities of sweetness, saltiness, sourness, bitterness, and pleasantness associated with each shape were compared for each group using a two-way factorial ANOVA with the awareness focus (PBE/NBE/control) and shape (Bouba/Kiki) as factors. In this experiment, five types of taste evaluations were measured. Therefore, each *p*-value was corrected by a factor of 5 using the Bonferroni correction, and a *p*-value of less than .05 was considered statistically significant.

### Results

#### Emotional Manipulation Check

**Figure 5.**
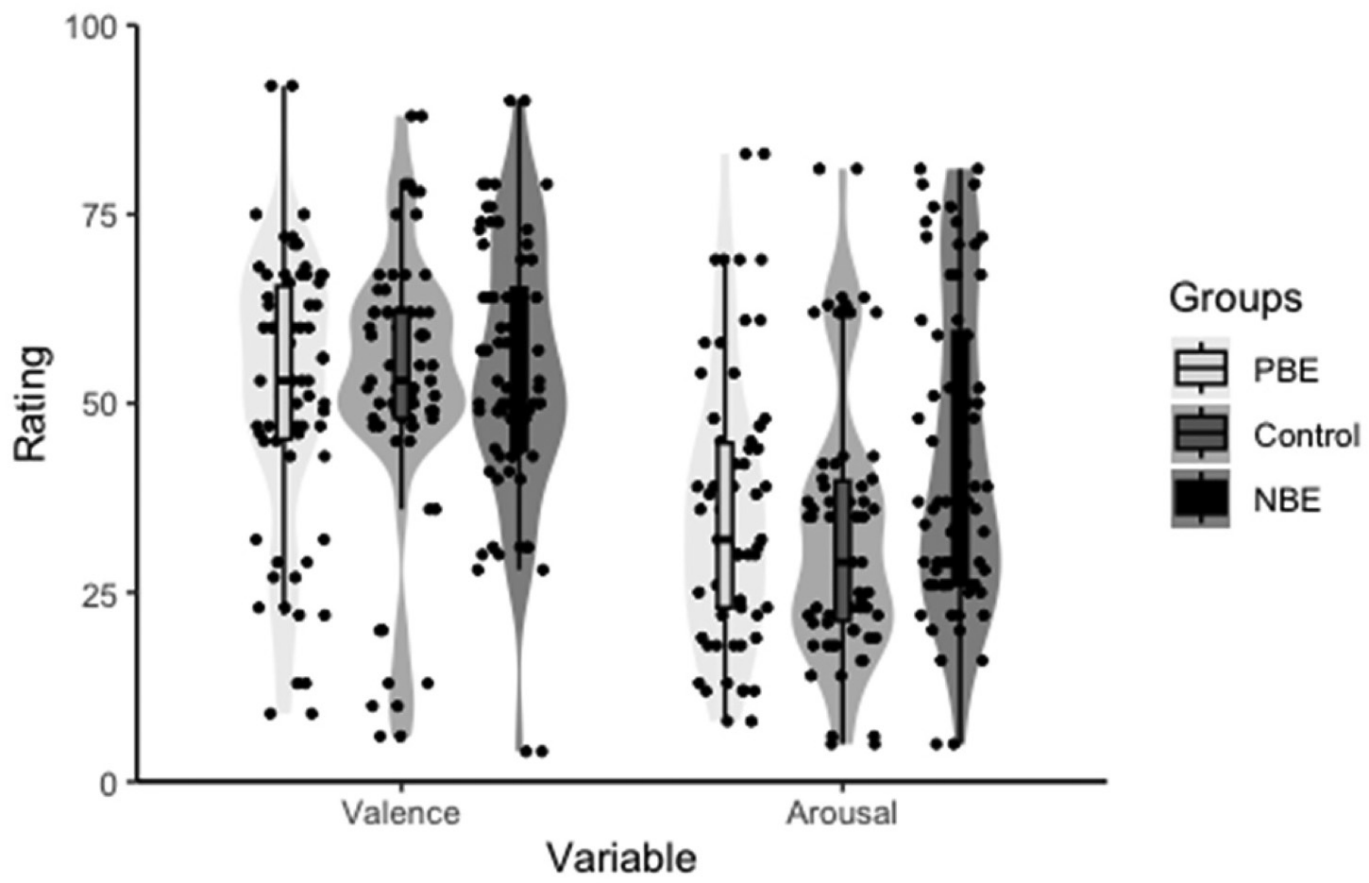
Violin plots and box-and-whisker plots for indicators of emotional states in Experiment 2. There were no significant differences between groups in valence ratings and arousal ratings.

The results of the one-way ANOVA confirmed that there was no significant influence of awareness focus on valence [*F* (2, 103) = 0.13, *p* = .8800, *η*^2^ = .05, BF_10_ = 0.03 (±0.01)] and arousal [*F* (2, 103) = 2.49, *p* = .0880, *η*^2^ = .05, BF_10_ = 0.26 (±0.01)] (Figure 5).

#### Taste Evaluations

**Figure 6.**
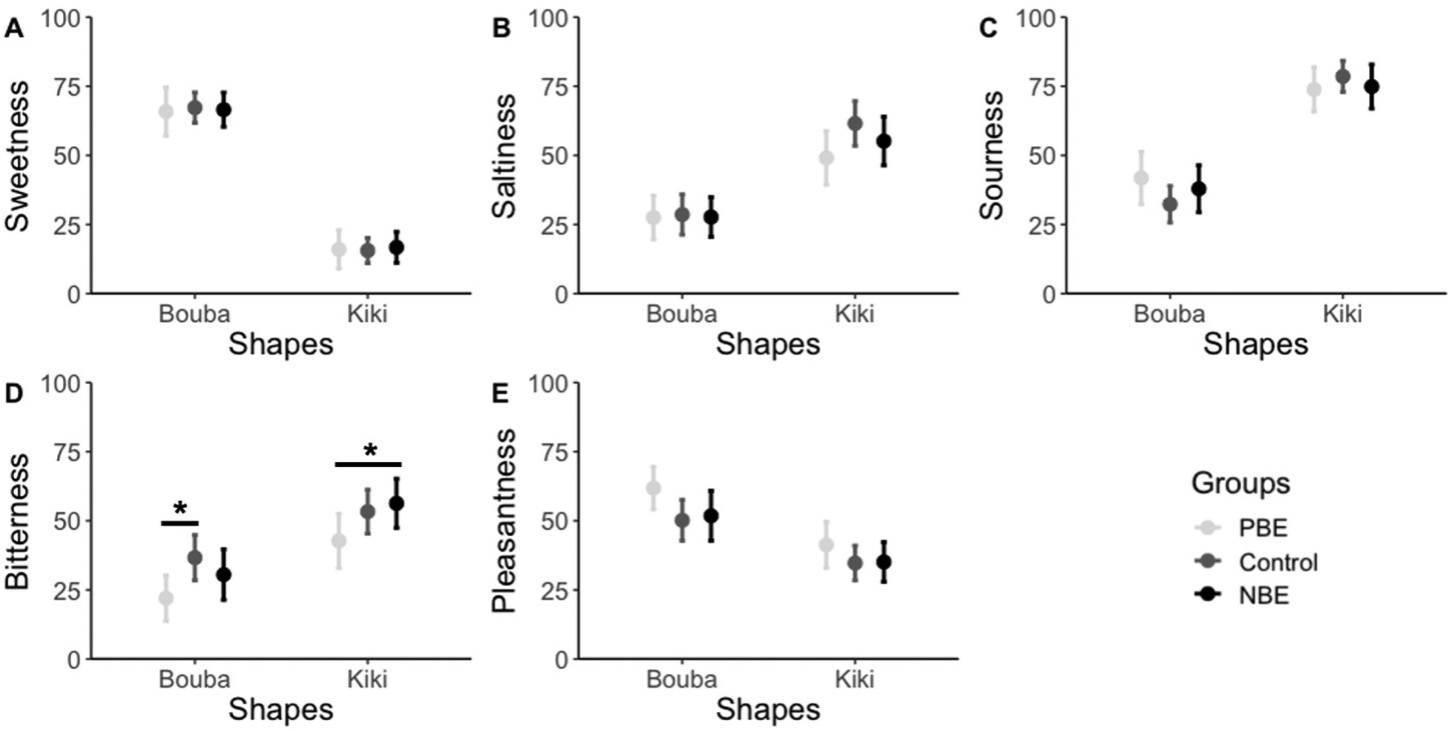
Intensity and 95% confidence interval predicted in Experiment 2. There were significant differences between the groups with bitterness. *** *p* < .001. ** *p* < .01. * *p* < .05. There were also significant differences between Bouba and Kiki ratings for each group (sweetness, saltiness, and sourness: all *p*-values .001; bitterness and pleasantness: all *p*-values .01).

A two-way ANOVA revealed that the main effect of shape was significant in all evaluations. Sweetness [*F* (1, 103) = 355.57, *p* < .0001, η*_p_^2^* = .78] and pleasantness [*F* (1, 103) = 38.38, *p* < .0001, η*_p_^2^* = .27] were rated significantly higher by Bouba than Kiki. Conversely, saltiness [*F* (1, 103) = 63.63, *p* < .0001, η*_p_^2^* = .38], sourness [*F* (1, 103) = 131.34, *p* < .0001, η*_p_^2^* = .56], and bitterness [*F* (1, 103) = 28.85, *p* < .0001, η*_p_^2^* = .22] were significantly higher in Kiki than in Bouba (Figure 6). In addition, there was a significant effect of awareness focus on bitterness [*F* (2,103) = 5.65, *p* = .0250, η*_p_^2^* = .10]. Multiple comparisons showed that control was significantly higher than PBE in Bouba (*p* = .0199) and NBE was significantly higher than PBE in Kiki (*p* = .0368). No other main effects or interactions were significant.

### Discussion

The results showed that the type of emotion to which awareness was directed had no significant influence on subsequent valence and arousal levels. Furthermore, each groups demonstrated nearly neutral emotional states. Therefore, it was confirmed that the taste evaluation data were influenced by emotion awareness immediately beforehand and not by differences in emotional states.

Moreover, the influence of the type of emotion awareness attended to beforehand was confirmed by bitterness evaluation. The awareness of negative emotion made the association between shape and bitterness stronger than when aware of non-emotion and significantly stronger than when aware of positive emotion. This result supports the findings of Experiment 1, which suggested that directing awareness to the negative emotion of anxiety leads to a stronger evaluation of bitterness. Notably, this phenomenon was observed even in the evaluation of visual stimuli that did not stimulate the taste receptors. This implies that cognitive information integration in taste perception processing might be involved in the amplification of bitterness due to negative emotions.

In a previous study, the bitterness evaluation of bitter chocolate and bitter green tea was influenced by the cross-modal effect of background colors. However, the bitterness evaluation of non-bitter chocolate and non-bitter green tea was unaffected (Sugimori & Kawasaki, 2022). Thus, the cross-modal influence on taste evaluation changes based on the characteristics of the subject stimulus. In the present study as well, no significant influence of awareness of negative emotion on bitterness evaluation was observed in the evaluation of Bouba, which has a low association with bitterness. In contrast, compared to the control, the awareness of positive emotion significantly led to a lower bitterness evaluation. Although the difference in the evaluation of Kiki was not significant, it displayed a lower bitterness evaluation than neutral. This suggests that the awareness of positive emotions might suppress bitterness evaluations, and this effect might be more pronounced in subject stimulus with lower bitterness.

Sweetness is associated with positive emotions (Liang et al., 2021; Zhou & Tse, 2020), but no sweetness enhancement due to the awareness of positive emotions has been observed in this experiment. This may be because the participants in this experiment were Japanese. In Japanese, “bitter” is always used as a negative expression, while the word “sweet” is used in a positive sense, such as “sweet love” and “sweet mask,” but it is also used in a negative sense, such as “sweet thinking,” which describes a lack of thinking, or “sweet talk,” which describes deceiving people with clever words. Hence, the correspondence between sweetness and positivity may be weaker than in other countries or regions. Therefore, the positive effects that the participants were aware of immediately before the evaluation may not have been reflected in their sweetness. Although a higher rating of pleasantness was associated with shapes in the PBE than in the NBE and control group, this effect was not statistically significant.

The two shapes used in Experiment 2, Bouba and Kiki, showed significant differences in taste quality and pleasantness ratings. As shown in previous studies (Deroy & Valentin, 2011; Salgado-Montejo et al., 2015, Velasco et al., 2016; Velasco et al., 2015; Velasco et al., 2016), Bouba, characterized by roundness, showed a stronger association with sweetness, whereas Kiki, characterized by angularity, showed a stronger association with saltiness, sourness, and bitterness. This investigation, therefore, confirmed that unique taste images could be perceived solely through visual cues, even in the absence of the direct stimulation of taste receptors. This suggests that the cross-modal effects of other sensory inputs on taste perception, as identified in prior research, may occur as a consequence of the integration of taste imagery into the cognitive processing of taste perception. Nevertheless, it was confirmed that two visual stimuli, each associated with a different taste, were selected for the experiment.

## General Discussion

In the present study, we considered two hypotheses regarding how emotional state influences taste perception. The first hypothesis posits that an awareness of emotional state could influence taste in cognitive processing, similar to information perceived in other modalities. The second hypothesis suggests that evoked emotional state modulates taste perception at the unconscious level. In this case, it is presumed that the evocation of this emotional state influences taste perception at the peripheral level, which is mediated by physiological changes. We conducted two experiments to validate these hypotheses. In Experiment 1, we investigated the influence of anxiety on taste perception when participants consumed two different types of coffee from the perspective of awareness toward the anxiety evoked. In Experiment 2, we further examined the influence of directing awareness toward negative emotions on the conceptual evaluation of taste quality without actual taste stimulation to reinforce the findings of Experiment 1.

The results revealed at least two patterns regarding the influence of anxiety, a negative emotion, on taste perception. The first pattern is an enhancement of bitterness when individuals focus on the negative emotions of anxiety rather than on inducing anxiety. This phenomenon was initially observed in Experiment 1, in which taste perception of actual taste stimuli was measured following emotional manipulation of awareness toward anxiety. Additionally, in Experiment 2, taste evaluations were measured in response to visual stimuli without taste stimulation after manipulating the awareness toward positive/negative emotions without emotional manipulation, which further supported this finding. The cross-modal influence of other modalities on taste perception may be due to the integration of the affective components induced by the stimulation of other modalities with taste information (Liang et al., 2020). Similarly, the affective component induced by the awareness of one's own internal information, the emotional state, may influence taste perception.　

The second pattern is the suppression of sweetness intensity, irrespective of awareness of anxiety, when individuals experience anxiety. This finding aligns with previous research demonstrating that the induction of negative emotions suppresses sweetness intensity (Al’Absi et al., 2012; Desira et al., 2020; Dess & Edelheit, 1998; Nakagawa et al., 1996; Noel & Dando, 2015; Zushi et al., 2021). As the influence of anxiety induction itself was observed regardless of awareness focus, this may be attributed to the physiological changes caused by anxiety. Previous studies have reported that heightened cortisol reactivity due to stress promotes the consumption of sweet foods (Epel et al., 2001) or snacks (Newman et al., 2007). Additionally, a previous study in mouse models revealed that glucocorticoid receptors are present in taste buds and are specifically localized to Tas1r3, the taste receptor subunit for sweet and umami tastes, and further showed that restraint stress induces their localization to the nucleus (Parker et al., 2014). Considering that cortisol responds to stress, the cortisol response induced by anxiety may mediate changes in sweetness perception. However, further investigations are necessary to verify this possibility.

This study had several limitations. First, the perceived saltiness intensity was low for both stimuli in Experiment 1 due to the characteristics of the stimuli. Therefore, we did not observe any perceptual changes due to the emotional states. However, trait anxiety also influences salt thresholds (Ileri-Gurel et al., 2013). It may therefore be worthwhile to consider taste quality in future studies. Second, we fixed the order of the relaxation and anxiety conditions in Experiment 1. This was due to the unclear duration of the effect of the anxiety condition on taste and our inability to control the state of each participant prior to their arrival at the lab. Hence, as a control condition, we conducted a relaxation condition in the first trial. Consequently, order effects may have influenced the results of this study. Nevertheless, sweetness was suppressed in the anxiety trial in both the EG and NEG, whereas bitterness was only enhanced in the anxiety trial in the EG. Furthermore, a significant difference was observed in the ratings of bitterness intensity under the anxiety condition between the EG and NEG, which followed the same fixed order as the relaxation and anxiety conditions. Therefore, it can be concluded that the phenomena observed in this study are not solely due to order effects. Third, this study was conducted on Japanese individuals. Although we have mentioned the possibility that metaphorical relationships may be reflected in taste perception, it is also possible that different results may be obtained when experiments are conducted with participants from different countries or regions. Fourth, this study did not account for the emotional susceptibility of the participants. In Experiment 1, while we imposed restrictions on BMI and food consumption prior to the experiment, we did not consider emotional traits like anxiety sensitivity (Taylor et al., 2007). However, previous studies suggest that individuals with high cortisol reactivity tend to consume more sweet and fatty foods under stress (Epel et al., 2001; Newman et al., 2007). This hints that taste perception might also be influenced by such emotion-related individual characteristics. Future study should not only assess emotional states but also measure emotional traits such as cortisol reactivity and anxiety sensitivity and examine their relationship to changes in taste perception. Finally, taste evaluations in both experiments were measured using the participants’ subjective evaluations. Therefore, the observations in this study are limited to a final evaluation of taste perception. Currently, the physiological and cognitive mechanisms by which emotional states influence taste perception are poorly understood. In the future, it will be necessary to conduct research to elucidate this mechanism from the perspective of taste receptor responses, hormones, and brain activity.

In conclusion, this is the first study to investigate the influence of emotional state on taste perception from the perspective of emotional awareness. The results of this study suggest that the mechanism by which emotional states influence taste can vary depending on taste quality. Sweetness, thought to be important for detecting energy sources, was found to have a reduced perception intensity when anxiety was induced. Bitterness, thought to be related to the avoidance of harmful substances, showed an enhancement in evaluation, not by the inducement of anxiety itself, but rather by the awareness directed toward anxiety or negative experiences. This suggests that the influence of emotional states on taste perception varies depending on their biological significance.

## References

[bibr1-20416695231216370] Al'Absi M. Nakajima M. Hooker S. Wittmers L. Cragin T. (2012). Exposure to acute stress is associated with attenuated sweet taste. Psychophysiology, 49(1), 96–103. 10.1111/j.1469-8986.2011.01289.x 22091733 PMC3240721

[bibr2-20416695231216370] Bartoshuk L. M. Duffy V. B. Hayes J. E. Moskowitz H. R. Snyder D. J. (2006). Psychophysics of sweet and fat perception in obesity: Problems, solutions and new perspectives. Philosophical Transactions of the Royal Society B: Biological Sciences, 361(1471), 1137–1148. 10.1098/rstb.2006.1853 PMC1642698 16815797

[bibr3-20416695231216370] Bates D. Mächler M. Bolker B. Walker S. (2015). Fitting linear mixed-effects models using lme4. Journal of Statistical Software, 67(1), 1–48. 10.18637/jss.v067.i01

[bibr4-20416695231216370] Biggs L. Juravle G. Spence C. (2016). Haptic exploration of plateware alters the perceived texture and taste of food. Food Quality and Preference, 50, 129–134. 10.1016/j.foodqual.2016.02.007

[bibr5-20416695231216370] Blossfeld I. Collins A. Boland S. Baixauli R. Kiely M. Delahunty C. (2007). Relationships between acceptance of sour taste and fruit intakes in 18-month-old infants. British Journal of Nutrition, 98(5), 1084–1091. 10.1017/S0007114507749231 17521470

[bibr6-20416695231216370] Bonfils P. Avan P. Faulcon P. Malinvaud D. (2005). Distorted odorant perception: Analysis of a series of 56 patients with parosmia. Archives of Otolaryngology–Head & Neck Surgery, 131(2), 107–112. 10.1001/archotol.131.2.107 15723940

[bibr7-20416695231216370] Bravo-Moncayo L. Reinoso-Carvalho F. Velasco C. (2020). The effects of noise control in coffee tasting experiences. Food Quality and Preference, 86, 104020. 10.1016/j.foodqual.2020.104020

[bibr8-20416695231216370] Briguglio G. Teodoro M. Italia S. Verduci F. Pollicino M. Coco M. De Vita A. Micali E. Alibrandi A. Lembo G. Costa C. Fenga C. (2021). Salivary biomarkers and work-related stress in night shift workers. International Journal of Environmental Research and Public Health, 18(6), 3184. 10.3390/ijerph18063184 33808679 PMC8003447

[bibr9-20416695231216370] Chojnowska S. Ptaszyńska-Sarosiek I. Kępka A. Knaś M. Waszkiewicz N. (2021). Salivary biomarkers of stress, anxiety and depression. Journal of Clinical Medicine, 10(3), 517. 10.3390/jcm10030517 33535653 PMC7867141

[bibr10-20416695231216370] Cartwright M. Wardle J. Steggles N. Simon A. E. Croker H. Jarvis M. J. (2003). Stress and dietary practices in adolescents. Health Psychology, 22(4), 362. 10.1037/0278-6133.22.4.362 12940392

[bibr11-20416695231216370] Demattè M. L. Pojer N. Endrizzi I. Corollaro M. L. Betta E. Aprea E. Charles M. Biasioli F. Zampini M. Gasperi F. (2014). Effects of the sound of the bite on apple perceived crispness and hardness. Food Quality and Preference, 38, 58–64. 10.1016/j.foodqual.2014.05.009

[bibr12-20416695231216370] Deroy O. Valentin D. (2011). Tasting liquid shapes: Investigating the sensory basis of cross-modal correspondences. Chemosensory Perception, 4, 80–90. 10.1007/s12078-011-9097-1

[bibr13-20416695231216370] Desira B. Watson S. Van Doorn G. Timora J. Spence C. (2020). Happy hour? A preliminary study of the effect of induced joviality and sadness on beer perception. Beverages, 6(2), 35. 10.3390/beverages6020035

[bibr14-20416695231216370] Dess N. K. Edelheit D. (1998). The bitter with the sweet: The taste/stress/temperament nexus. Biological Psychology, 48(2), 103–119. 10.1016/S0301-0511(98)00014-3 9700013

[bibr15-20416695231216370] Di Lorenzo P. M. (2021). Taste in the brain is encoded by sensorimotor state changes. Current Opinion in Physiology, 20, 39–45. 10.1016/j.cophys.2020.12.003

[bibr16-20416695231216370] Drewnowski A. Gomez-Carneros C. (2000). Bitter taste, phytonutrients, and the consumer: A review. The American Journal of Clinical Nutrition, 72(6), 1424–1435. 10.1093/ajcn/72.6.1424 11101467

[bibr17-20416695231216370] Dunn O. J. (1961). Multiple comparisons among means. Journal of the American Statistical Association, 56(293), 52–64. 10.1080/01621459.1961.10482090

[bibr18-20416695231216370] Epel E. Lapidus R. McEwen B. Brownell K. (2001). Stress may add bite to appetite in women: A laboratory study of stress-induced cortisol and eating behavior. Psychoneuroendocrinology, 26(1), 37–49. 10.1016/S0306-4530(00)00035-4 11070333

[bibr19-20416695231216370] Faul F. Erdfelder E. Buchner A. Lang A.-G. (2009). Statistical power analyses using g* power 3.1: Tests for correlation and regression analyses. Behavior Research Methods, 41(4), 1149–1160. 10.3758/BRM.41.4.1149 19897823

[bibr20-20416695231216370] Ganchrow J. R. Steiner J. E. Daher M. (1983). Neonatal facial expressions in response to different qualities and intensities of gustatory stimuli. Infant Behavior and Development, 6(4), 473–484. 10.1016/S0163-6383(83)90301-6

[bibr21-20416695231216370] Hardikar S. Höchenberger R. Villringer A. Ohla K. (2017). Higher sensitivity to sweet and salty taste in obese compared to lean individuals. Appetite, 111, 158–165. 10.1016/j.appet.2016.12.017 27988366

[bibr22-20416695231216370] Herman C. P. Polivy J. (1975). Anxiety, restraint, and eating behavior. Journal of Abnormal Psychology, 84(6), 666. 10.1037/0021-843X.84.6.666 1194527

[bibr23-20416695231216370] Hidano N. Fukuhara M. Iwawaki M. Soga S. Spielberger C. (2000). State-trait anxiety inventory-form JYZ. Japan UNI Agency. in Japanese.

[bibr24-20416695231216370] Ichimura F. Motoki K. Matsushita K. Ariga A. (2023). The tactile thickness of the lip and weight of a glass can modulate sensory perception of tea beverage. Food and Humanity, 1, 180–187. 10.1016/j.foohum.2023.05.011

[bibr25-20416695231216370] Ileri-Gurel E. Pehlivanoglu B. Dogan M. (2013). Effect of acute stress on taste perception: In relation with baseline anxiety level and body weight. Chemical Senses, 38(1), 27–34. 10.1093/chemse/bjs075 22944612

[bibr26-20416695231216370] Klein L. C. Bennett J. M. Whetzel C. A. Granger D. A. Ritter F. E. (2010). Caffeine and stress alter salivary *α*-amylase activity in young men. Human Psychopharmacology: Clinical and Experimental, 25(5), 359–367. 10.1002/hup.1126 20589924

[bibr27-20416695231216370] Kuznetsova A. Brockhoff P. B. Christensen R. (2017). Lmertest package: Tests in linear mixed effects models. Journal of Statistical Software, 82(13), 1–26. 10.18637/jss.v082.i13

[bibr28-20416695231216370] Liang P. Jiang J. Y. Liu Q. Zhang S. L. Yang H. J. (2020). Mechanism of cross-modal information influencing taste. Current Medical Science, 40(3), 474–479. 10.1007/s11596-020-2206-0 32681252

[bibr29-20416695231216370] Liang P. Jiang J. Y. Wei L. Ding Q. (2021). Direct mapping of affective pictures and taste words. Food Quality and Preference, 89, 104151. 10.1016/j.foodqual.2020.104151

[bibr30-20416695231216370] Liem D. G. Mennella J. A. (2003). Heightened sour preferences during childhood. Chemical Senses, 28(2), 173–180. 10.1093/chemse/28.2.173 12588738 PMC2789429

[bibr31-20416695231216370] Lindemann B. (2001). Receptors and transduction in taste. Nature, 413(6852), 219–225. 10.1038/35093032 11557991

[bibr32-20416695231216370] Macht M. (2008). How emotions affect eating: A five-way model. Appetite, 50(1), 1–11. 10.1016/j.appet.2007.07.002 17707947

[bibr34-20416695231216370] Mennella J. A. Beauchamp G. K. (1998). Early flavor experiences: Research update. Nutrition Reviews, 56(7), 205–211. 10.1111/j.1753-4887.1998.tb01749.x 9697386

[bibr35-20416695231216370] Moir H. (1936). Some observations on the appreciation of flavour in foodstuffs. Journal of the Society of Chemical Industry, 55(8), 145–148. 10.1002/jctb.5000550803

[bibr36-20416695231216370] Nakagawa M. Mizuma K. Inui T. (1996). Changes in taste perception following mental or physical stress. Chemical Senses, 21(2), 195–200. 10.1093/chemse/21.2.195 8670698

[bibr37-20416695231216370] Nakata H . (Director). (2002). “*Honogurai Mizu no Soko kara*” [“Dark water”][Movie]. KADOKAWA.

[bibr38-20416695231216370] Newman E. O'Connor D. B. Conner M. (2007). Daily hassles and eating behaviour: The role of cortisol reactivity status. Psychoneuroendocrinology, 32(2), 125–132. 10.1016/j.psyneuen.2006.11.006 17198744

[bibr39-20416695231216370] Nissim I. Dagan-Wiener A. Niv M. Y. (2017). The taste of toxicity: A quantitative analysis of bitter and toxic molecules. IUBMB life, 69(12), 938–946. 10.1002/iub.1694 29130618

[bibr40-20416695231216370] Noel C. Dando R. (2015). The effect of emotional state on taste perception. Appetite, 95, 89–95. 10.1016/j.appet.2015.06.003 26122754

[bibr41-20416695231216370] Oliver G. Wardle J. (1999). Perceived effects of stress on food choice. Physiology & Behavior, 66(3), 511–515. 10.1016/s0031-9384(98)00322-9 10357442

[bibr42-20416695231216370] Oliver G. Wardle J. Gibson E. L. (2000). Stress and food choice: A laboratory study. Psychosomatic Medicine, 62(6), 853–865. 10.1097/00006842-200011000-00016 11139006

[bibr43-20416695231216370] Overberg J. Hummel T. Krude H. Wiegand S. (2012). Differences in taste sensitivity between obese and non-obese children and adolescents. Archives of Disease in Childhood, 97(12), 1048–1052. 10.1136/archdischild-2011-301189 22995095

[bibr44-20416695231216370] Parker M. R. Feng D. Chamuris B. Margolskee R. F. (2014). Expression and nuclear translocation of glucocorticoid receptors in type 2 taste receptor cells. Neuroscience Letters, 571, 72–77. 10.1016/j.neulet.2014.04.047 24814581 PMC4126247

[bibr45-20416695231216370] Pasquet P. Laure Frelut M. Simmen B. Marcel Hladik C. Monneuse M. O. (2007). Taste perception in massively obese and in non-obese adolescents. International Journal of Pediatric Obesity, 2(4), 242–248. 10.1080/17477160701440521 17852551

[bibr46-20416695231216370] Piqueras-Fiszman B. Alcaide J. Roura E. Spence C. (2012). Is it the plate or is it the food? Assessing the influence of the color (black or white) and shape of the plate on the perception of the food placed on it. Food Quality and Preference, 24(1), 205–208. 10.1016/j.foodqual.2011.08.011

[bibr47-20416695231216370] Platte P. Herbert C. Pauli P. Breslin P. A. (2013). Oral perceptions of fat and taste stimuli are modulated by affect and mood induction. PLoS One, 8(6), e65006. 10.1371/journal.pone.0065006 PMC3673997 23755167

[bibr48-20416695231216370] Poquérusse J. Azhari A. Setoh P. Cainelli S. Ripoli C. Venuti P. Esposito G. (2018). Salivary *α*-amylase as a marker of stress reduction in individuals with intellectual disability and autism in response to occupational and music therapy. Journal of Intellectual Disability Research, 62(2), 156–163. 10.1111/jir.12453 29159888

[bibr49-20416695231216370] Rahne T. Köppke R. Nehring M. Plontke S. K. Fischer H. G. (2018). Does ambient noise or hypobaric atmosphere influence olfactory and gustatory function? PLoS One, 13(1), e0190837. 10.1371/journal.pone.0190837 PMC5784903 29370217

[bibr50-20416695231216370] Ramachandran V. S. Hubbard E. M. (2001). Synaesthesia -a window into perception, thought and language. Journal of Consciousness Studies, 8(12), 3–34.

[bibr51-20416695231216370] R Core Team. (2020). R: A language and environment for statistical computing. r foundation for statistical computing, Vienna, Austria. http://www.r-project.org/index.html

[bibr52-20416695231216370] Reinoso-Carvalho F. Dakduk S. Wagemans J. Spence C. (2019). Not just another pint! the role of emotion induced by music on the consumer’s tasting experience. Multisensory Research, 32(4-5), 367–400. 10.1163/22134808-20191374 31059486

[bibr53-20416695231216370] Reinoso-Carvalho F. Gunn L. H. Horst E. T. Spence C. (2020). Blending emotions and cross-modality in sonic seasoning: Towards greater applicability in the design of multisensory food experiences. Foods (Basel, Switzerland), 9(12), 1876. 10.3390/foods9121876 33348626 PMC7766860

[bibr54-20416695231216370] Salgado-Montejo A. Alvarado J. A. Velasco C. Salgado C. J. Hasse K. Spence C. (2015). The sweetest thing: The influence of angularity, symmetry, and the number of elements on shape-valence and shape-taste matches. Frontiers in Psychology, 6, 1382. 10.3389/fpsyg.2015.01382 26441757 PMC4569812

[bibr55-20416695231216370] Simchen U. Koebnick C. Hoyer S. Issanchou S. Zunft H.-J. (2006). Odour and taste sensitivity is associated with body weight and extent of misreporting of body weight. European Journal of Clinical Nutrition, 60(6), 698–705. 10.1038/sj.ejcn.1602371 16435003

[bibr56-20416695231216370] Small D. M. (2012). Flavor is in the brain. Physiology & Behavior, 107(4), 540–552. 10.1016/j.physbeh.2012.04.011 22542991

[bibr57-20416695231216370] Small D. M. Prescott J. (2005). Odor/taste integration and the perception of flavor. Experimental Brain Research, 166(3), 345–357. 10.1007/s00221-005-2376-9 16028032

[bibr58-20416695231216370] Smit H. Rogers P. (2000). Effects of low doses of caffeine on cognitive performance, mood and thirst in low and higher caffeine consumers. Psychopharmacology, 152(2), 167–173. 10.1007/s002130000506 11057520

[bibr59-20416695231216370] Smith B. A. Fillion T. J. Blass E. M. (1990). Orally mediated sources of calming in 1-to 3-day-old human infants. Developmental Psychology, 26(5), 731. 10.1037/0012-1649.26.5.731

[bibr60-20416695231216370] Spence C. (2020). Assessing the role of emotional mediation in explaining crossmodal correspondences involving musical stimuli. Multisensory Research, 33(1), 1–29. 10.1163/22134808-20191469 31648195

[bibr61-20416695231216370] Spielberger C. Goruch R. Lushene R. Vagg P. Jacobs G. (1983). Manual for the state-trait inventory stai (form y). *Mind Garden, Palo Alto, CA, USA*.

[bibr62-20416695231216370] Stafford L. D. Fernandes M. Agobiani E. (2012). Effects of noise and distraction on alcohol perception. Food Quality and Preference, 24(1), 218–224. 10.1016/j.foodqual.2011.10.012

[bibr63-20416695231216370] Stewart P. C. Goss E. (2013). Plate shape and colour interact to influence taste and quality judgments. Flavour, 2(1), 1–9. 10.1186/2044-7248-2-27

[bibr64-20416695231216370] Sugimori E. Kawasaki Y. (2022). Cross-modal correspondence between visual information and taste perception of bitter foods and drinks. Food Quality and Preference, 98, 104539. 10.1016/j.foodqual.2022.104539

[bibr65-20416695231216370] Tahara A . (Video producer). (2010). “Sekai no Shasō Kara” (DVD book No.32) [“Next Journey”][TV series episode]. Asahi Simbun Publications.

[bibr66-20416695231216370] Takahira M. (1998). Construction of a scale of life events in interpersonal and achievement domains for undergraduate students. Japanese Journal of Social Psychology, 14(1), 12–24. 10.14966/jssp.KJ00004622673

[bibr67-20416695231216370] Taylor S. Zvolensky M. J. Cox B. J. Deacon B. Heimberg R. G. Ledley D. R. Abramowitz J. S. Holaway R. M. Sandin B. Stewart S. H. Coles M. Eng W. Daly E. S. Arrindell W. A. Bouvard M. Cardenas S. J. (2007). Robust dimensions of anxiety sensitivity: Development and initial validation of the Anxiety Sensitivity Index-3. Psychological Assessment, 19(2), 176–188. 10.1037/1040-3590.19.2.176 17563199

[bibr68-20416695231216370] Tu Y. Yang Z. Ma C. (2015). Touching tastes: The haptic perception transfer of liquid food packaging materials. Food Quality and Preference, 39, 124–130. 10.1016/j.foodqual.2014.07.001

[bibr69-20416695231216370] Velasco C. Woods A. T. Hyndman S. Spence C. (2015). The taste of typeface. i-Perception, 6(4), 2041669515593040. 10.1177/2041669515593040 PMC4934647 27433316

[bibr70-20416695231216370] Velasco C. Woods A. T. Marks L. E. Cheok A. D. Spence C. (2016a). The semantic basis of taste-shape associations. PeerJ, 4, e1644. 10.7717/peerj.1644 PMC4783761 26966646

[bibr71-20416695231216370] Velasco C. Woods A. T. Petit O. Cheok A. D. Spence C. (2016b). Crossmodal correspondences between taste and shape, and their implications for product packaging: A review. Food Quality and Preference, 52, 17–26. 10.1016/j.foodqual.2016.03.005

[bibr72-20416695231216370] Wang Q. J. Spence C. (2016). Striking a sour note’: Assessing the influence of consonant and dissonant music on taste perception. Multisensory Research, 29(1-3), 195–208. 10.1163/22134808-00002505 27311296

[bibr73-20416695231216370] Wardle J. Steptoe A. Oliver G. Lipsey Z. (2000). Stress, dietary restraint and food intake. Journal of Psychosomatic Research, 48(2), 195–202. 10.1016/S0022-3999(00)00076-3 10719137

[bibr74-20416695231216370] Woods A. T. Poliakoff E. Lloyd D. M. Kuenzel J. Hodson R. Gonda H. Thomas A. (2011). Effect of background noise on food perception. Food Quality and Preference, 22(1), 42–47. 10.1016/j.foodqual.2010.07.003

[bibr75-20416695231216370] Yanai H. Kashiwagi S. Kokusho R. (1987). Construction of a new personality inventory by means of factor analysis based on promax rotation. Japanese Journal of Psychology, 58(3), 158–165. 10.4992/jjpsy.58.158 3450905

[bibr76-20416695231216370] Zampini M. Spence C. (2004). The role of auditory cues in modulating the perceived crispness and staleness of potato chips. Journal of Sensory Studies, 19(5), 347–363. 10.1111/j.1745-459x.2004.080403.x

[bibr77-20416695231216370] Zhou Y. Tse C.-S. (2020). The taste of emotion: Metaphoric association between taste words and emotion/emotion-laden words. Frontiers in Psychology, 11, 986. 10.3389/fpsyg.2020.00986 32581914 PMC7290244

[bibr78-20416695231216370] Zushi N. Ogawa M. Ayabe-Kanamura S. (2021). Fear reduces perceived sweetness: Changes in the perception of taste due to emotional state. SAGE Open, 11(1), 2158244021989318. 10.1177/215824402198931

